# PAK2 is necessary for myelination in the peripheral nervous system

**DOI:** 10.1093/brain/awad413

**Published:** 2023-12-11

**Authors:** Bo Hu, Daniel Moiseev, Isabella Schena, Bulat Faezov, Roland Dunbrack, Jonathan Chernoff, Jun Li

**Affiliations:** Department of Neurology, Houston Methodist Research Institute, Houston, TX 77030, USA; Department of Neurology, Wayne State University School of Medicine, Detroit, MI 48201, USA; Department of Neurology, Houston Methodist Research Institute, Houston, TX 77030, USA; Cancer Biology, Fox Chase Cancer Center, Philadelphia, PA 19111, USA; Fundamental Medicine and Biology, Kazan Federal University, Kazan 420008, Russia; Cancer Biology, Fox Chase Cancer Center, Philadelphia, PA 19111, USA; Cancer Biology, Fox Chase Cancer Center, Philadelphia, PA 19111, USA; Department of Neurology, Houston Methodist Research Institute, Houston, TX 77030, USA

**Keywords:** *Pak2* knock-out mouse, peripheral nerve, myelination, Schwann cells, GTPases Rac1/Cdc42, myelin lipids

## Abstract

Myelination enables electrical impulses to propagate on axons at the highest speed, encoding essential life functions. The Rho family GTPases, RAC1 and CDC42, have been shown to critically regulate Schwann cell myelination. P21-activated kinase 2 (PAK2) is an effector of RAC1/CDC42, but its specific role in myelination remains undetermined. We produced a Schwann cell-specific knockout mouse of *Pak2* (*scPak2*^−/−^) to evaluate PAK2’s role in myelination.

Deletion of *Pak2*, specifically in mouse Schwann cells, resulted in severe hypomyelination, slowed nerve conduction velocity and behaviour dysfunctions in the *scPak2*^−/−^ peripheral nerve. Many Schwann cells in *scPak2*^−/−^ sciatic nerves were arrested at the stage of axonal sorting. These abnormalities were rescued by reintroducing *Pak2*, but not the kinase-dead mutation of *Pak2*, via lentivirus delivery to *scPak2*^−/−^ Schwann cells *in vivo*. Moreover, ablation of *Pak2* in Schwann cells blocked the promyelinating effect driven by neuregulin-1, prion protein and inactivated RAC1/CDC42. Conversely, the ablation of *Pak2* in neurons exhibited no phenotype. Such PAK2 activity can also be either enhanced or inhibited by different myelin lipids.

We have identified a novel promyelinating factor, PAK2, that acts as a critical convergence point for multiple promyelinating signalling pathways. The promyelination by PAK2 is Schwann cell-autonomous. Myelin lipids, identified as inhibitors or activators of PAK2, may be utilized to develop therapies for repairing abnormal myelin in peripheral neuropathies.

## Teaser

Hu *et al.* generate mice with a conditional deletion of P21-activated kinase 2 (*Pak2*) in Schwann cells, and show that Pak2 acts as a critical convergence point for multiple promyelinating signalling pathways. Loss of Pak2 from Schwann cells results in severe hypomyelination and reduced nerve conduction velocity.

## Introduction

Myelin, the multilayered lipid-rich-membrane sheath that wraps axons concentrically, is essential for transmitting electrical impulses in the nervous system. These impulses encode signals for executing numerous functions conserved in mammals, vertebrates and invertebrates. Myelinating Schwann cells integrate axonal signals to regulate myelin formation during development.^1^ For instance, axon-derived type III neuregulin-1 (Nrg1) binds to ErbB2/3 receptors on Schwann cells to activate small GTPases RAC1/CDC42 and promote myelination.^2^ Prion protein (PrP^C^) from axon interacts with G protein-coupled receptors (GPCRs) on Schwann cells, leading to trimeric G protein complex activation, RAC1/CDC42 activation, and cAMP-driven myelination.^3,4^ Thus, cyclic adenosine monophosphate (cAMP) in Schwann cells is an essential second messenger for promyelination.^5^ However, as an effector of RAC1/CDC42, how P21-activated kinase 2 (PAK2) plays a role in these promyelinating pathways is unclear.

PAKs, a family of serine/threonine protein kinases, are divided into two groups based on the homology of amino acid sequences: Group I (PAK1, PAK2 and PAK3); and Group II (PAK4, PAK5 and PAK6).^6,7^ PAKs are directly downstream to small GTPases, RAC1 and CDC42.^8^ While Group I PAK members share considerable homology, each of them still exhibits distinct functions.^7^*Pak1* and *Pak3* knockout mice present no or minimal phenotype, but *Pak2* knockout is embryonically lethal in mice.^9-11^ Because ablation of *Rac1* or *Cdc42* arrests Schwann cells in their early stage of myelination, and PAKs are major druggable effectors of the small GTPases, it is important to clarify if and how PAK2 affects myelination.^12-14^

We generated mice with a conditional deletion of *Pak2* in Schwann cells. Our results revealed that PAK2 activity is indispensable for myelination. Moreover, this promyelinating effect can be regulated by myelin lipids, which may provide potential mechanisms for curbing or facilitating PAK2’s effect in myelination.

## Materials and methods

### Mice

Mice carrying floxed *Pak2* alleles^15,16^ (*Pak2^f/f^*, C57BL/6J background) were crossed with myelin protein zero-cre (*Mpz-cre*) mice (Strain #017927, Jackson Laboratory) to delete *Pak2* in Schwann cells (*scPak2*^−/−^). In addition, *Pak2^f/f^* mice were bred with Synapsin I-cre (*Syn-cre*) mice (Strain #003966, Jackson Laboratory) to ablate *Pak2* in neurons (*nPak2*^−/−^). *Pak2^f/+^:Mpz^cre+^* and *Pak2^f/+^:Syn^cre+^* mice were backcrossed at least nine times onto the C57BL/6J background. Littermate *Pak2^f/f^* mice were used as controls. PCR analysis for genotyping *Pak2* and *Cre* alleles used the following primers: for *Pak2*, forward was 5′-ATCTTCCCAGGCTCCTGACT-3′ and reverse 5′-TGAAGCTGCATCAATCTATTCTG-3′; for *Mpz-cre* and *Syn-cre*, forward was 5′-ACCCTGTTACGTATAGCCGA-3′ and reverse 5′-CTCCGGTATTGAAACTCCAG-3′. Controls were age- and sex-matched littermates. However, when evaluating body weight, male and female mice were separated for comparison. Mice were maintained on a standard 12-h light/12-h dark cycle, *ad libitum* access to food and water, and housed in plastic cages with standard rodent bedding.

### Animal ethics statements

All animal experiments and procedures were reviewed and approved by the Institutional Animal Care and Use Committee (IACUC) at the Houston Methodist Research Institute and were performed in accordance with federal and institute guidelines and regulations for the care and use of experimental vertebrate animals. The authors complied with the ARRIVE guidelines for reporting.

### Rotarod test

This method was previously described.^17^ Mice were placed on an accelerating (0.1 rpm/s) rotarod (Columbus Instruments) that progressed from 16 to 28 rpm over 120 s. After positioning, the mice walked until they fell or finished the 120 s run. Each mouse received 2 days of training before the test. All mice participated in daily trials for 3 days, with a 30-min break separating each trial. The latency to fall was averaged for each mouse.

### Hindlimb clasping test

Mice were held by the tail 30 cm above a tabletop and video-recorded for 20 s. Abnormal postures, such as hindlimb clasping and sustained straining of paws, were documented. A researcher unaware of the genotype examined the videos and recorded the duration of clasping and paw straining.

### Nerve conduction studies

Mouse nerve conduction studies (NCS) were previously described.^9^ In brief, mice were anaesthetized with isoflurane and compound muscle action potential (CMAP) measurements were taken from the intrinsic foot muscles using needle electrodes. Stimulation electrodes were placed at the mouse’s sciatic notch and ankle, with recordings made on the paw. Supramaximal stimulations were used to elicit CMAP and the conduction velocity (CV) was calculated between distal and proximal stimulation sites.

### Morphometric analysis and electron microscopy

The morphometric analysis has been detailed previously.^18^ Semi-thin sections from Epon-embedded sciatic nerves were stained with toluidine blue and imaged using a Leica THUNDER microscope. We segmented nerve images based on a U-net architecture in the convoluted neural network (CNN) model.^18^ After learning from manually segmented nerve images, this model automatically segmented myelin sheaths from axons. Using the inner and outer areas of each nerve fibre, the program calculated both inner and outer radii through a designated formula. The radii led to computing the myelin thickness by subtracting the inner radius from the outer radius and dividing the result by two, as well as the g-ratio, representing the ratio of the inner radius to the outer radius. Axon density was assessed by dividing the number of axons by the area containing all captured nerve fibres.

Electron microscopic analysis of mouse sciatic nerves was performed as previously described.^19^ The ultrathin sections were stained with uranyl acetate and lead citrate. Imaging was carried out using a Zeiss Gemini 300 Scanning Electron Microscope.

### Animal surgery and intraneural injection

Green fluorescent protein (GFP)-only, GFP-WT-PAK2 and GFP-K278R-PAK2 lentivirus were purchased from Applied Biological Materials and injected into sciatic nerves using a published protocol.^20^ Pups at P3 were anaesthetized with isoflurane inhalation and positioned under a Leica stereomicroscope. The sciatic nerve was exposed surgically. A fine glass needle (Fivephoton Biochemicals) containing 2 μl of 10^10^ cfu/ml lentivirus particles mixed with 0.01% Fast Green was injected into the sciatic nerve using a micromanipulator until the nerves appeared green. The contralateral sciatic nerve, as a control, received a 2 μl PBS with 0.01% Fast Green injection. Once the injection was completed, the wound was closed with Histoacryl glue. At Day 30 post-injection, sciatic nerves were dissected for immunofluorescence staining.

### Immunofluorescence staining

This method was previously described.^9^ In brief, for teased nerve fibre staining, sciatic nerves were fixed in 4% paraformaldehyde (PFA) overnight and teased into individual fibres on glass slides. For paraffin nerve staining, sciatic nerves were fixed, embedded in paraffin and sectioned into 5 μm-thick slices. The slides were incubated with primary antibodies at 4°C overnight. Following a wash, the slides were incubated with secondary antibodies for 1 h. The stained slides were observed under a Leica THUNDER fluorescent microscope.

### Cell culture and cAMP measurement

Schwann cells were obtained from the sciatic nerves of 3-day-old *Pak2^f/f^* and *scPak2*^−/−^ mice. Schwann cells were immortalized using an *SV40-T* lentiviral vector (Applied Biological Materials Inc). Human wild-type *PAK2* and kinase-dead *PAK2-K278R* plasmids were transfected into *Pak2*^−/−^ Schwann cells to generate overexpressing wild-type PAK2 (*Pak2-OE*) and kinase-dead PAK2 (*Pak2-K278R*) cell clones.

The four Schwann cell lines, *Pak2*^+/+^, *Pak2*^−/−^, *Pak2-OE* and *Pak2-K278R*, were treated with PBS, Nrg1 (50 ng/ml, Cat. No. 377HB, R&D Systems) or synthetic flexible tail (residues 23–50) of PrP^C^ peptides^4^ (2 μM, GenScript) for 30 min. The cells were lysed using 0.1 M HCl lysis buffer. The following steps were carried out according to the manufacturer’s protocol (Enzo Life Sciences, Cat. No. ADI-900-066), which involved competition between the sample cAMP and a cAMP-alkaline phosphatase conjugate. cAMP levels were determined using a cAMP standard curve in the context of an ELISA-based assay.

### Nrg1 and PrP^C^ stimulation and GTPase pull-down assay

*Pak2* ^+/+^ and *Pak2*^−/−^ Schwann cells were plated onto poly-L-lysine (PLL)-coated 100 mm plates and grown in Dulbecco’s modified Eagle medium (DMEM)/F12 supplemented with 10% fetal bovine serum (FBS) until they reached 80% confluency. To induce serum and mitogen starvation, cells were washed twice with pre-warmed Hanks’ Balanced Salt Solution (HBSS) and incubated in DMEM/F12 containing 0.5% FBS (starvation medium) for 20 h. After the incubation, three replicates of each plate were treated for 1 h at 37°C and 5% CO_2_ using either starvation medium with a vehicle (PBS plus 0.02% FBS; mock medium), 50 ng/ml Nrg1, or 2 μM PrP^C^ peptide.

Following the stimulation period, cells were washed twice with PBS containing phosphatase inhibitors (Cat. No. 5872, Cell Signaling) and lysed with 1× lysis buffer. Total protein concentration was determined using the BCA assay (Prod. No. 23225, Thermo Scientific). The active GTPase pull-down assay was performed according to the manufacturer’s instructions for the active RAC1 detection kit (Cat. No. 8815, Cell Signaling) and active CDC42 detection kit (Cat. No. 8819, Cell Signaling), with samples treated with GTPγS (positive control) and GDP (negative control). The immunoprecipitated materials and total protein samples were then prepared for immunoblot analysis.

### Immunoblotting

This method has been described previously.^9^ Whole cells or chopped sciatic nerves were lysed in RIPA buffer (Cat. No. R0278, Sigma) with a proteinase/phosphatase inhibiter cocktail. Protein concentration was measured using a BCA assay. Equal volumes of proteins were separated using SDS-PAGE gels and transferred to a polyvinylidene fluoride (PVDF) membrane. After blocking, primary and secondary antibodies were used to detect target proteins. Chemiluminescence (Cat. No. NEL103001, Perkin Elmer) was used for detection and ImageJ software quantified the protein bands.

### Antibodies and materials

PAK1 (Cat. No. 2602), PAK2 (Cat. No. 2608), PAK3 (Cat. No. 2609), PAK4 (Cat. No. 52694), Phospho-PAK2 (Ser20) (Cat. No. 2607), Phospho-PAK1 (Ser144)/PAK2 (Ser141) (Cat. No. 2606), Phospho-PAK1 (Thr423)/PAK2 (Thr402) (Cat. No. 2601), β-Tubulin (Cat. No. 2128) and β-actin (Cat. No. 4970) antibodies were from Cell Signaling Technology. PAK5 (Cat. No. ab110069), PAK6 (Cat. No. ab154752) and GAPDH (Cat. No. ab9485) antibodies were from Abcam. MBP (Cat. No. MAB386) and Neurofilament 200 (Cat. No. MAB5266) antibodies were from Millipore. Sphingomyelin (SM, #860062), L-α-phosphatidylinositol (PI, #840042), Ganglioside G_M3_ (GM3, #860058), cholesterol (CHOL, #700100), 18:1 cholesteryl oleate (CHOL-E, #700269), L-α-phosphatidylserine (PS, #840032) and C18:1 Galactosyl (β) Ceramide (G-Cer, #860596) were from Avanti Polar Lipids. *N*-hexanoyl-D-sphingosine (C6-Cer, #H6524) and D-sphingosine (SPH, #S7049) were from Sigma Aldrich.

### Protein structure analysis

AlphaFold-Multimer v3 (https://doi.org/10.1101/2021.10.04.463034) was run locally with default parameters with Nvidia 24 Gbyte GPUs. Uniprot90 was used as the sequence database for the generation of multiple sequence alignments (MSAs), which are input features to AlphaFold-Multimer. No template structures from the Protein Data Bank (PDB) were used. Twenty-five models of each complex were made (5 seeds × 5 AlphaFold-Multimer parameter sets). Structures were visualized with PyMOL.

### Statistical analysis

Statistical analysis was conducted using GraphPad Prism software version 9.0 and data were represented as the mean ± standard deviation (SD). *P*-values were derived from either a two-tailed Student’s *t*-test or repeated-measures ANOVA. A *P*-value < 0.05 was deemed statistically significant.

## Results

### PAK2 is highly expressed during the development of the peripheral nerves

**Figure 1:**
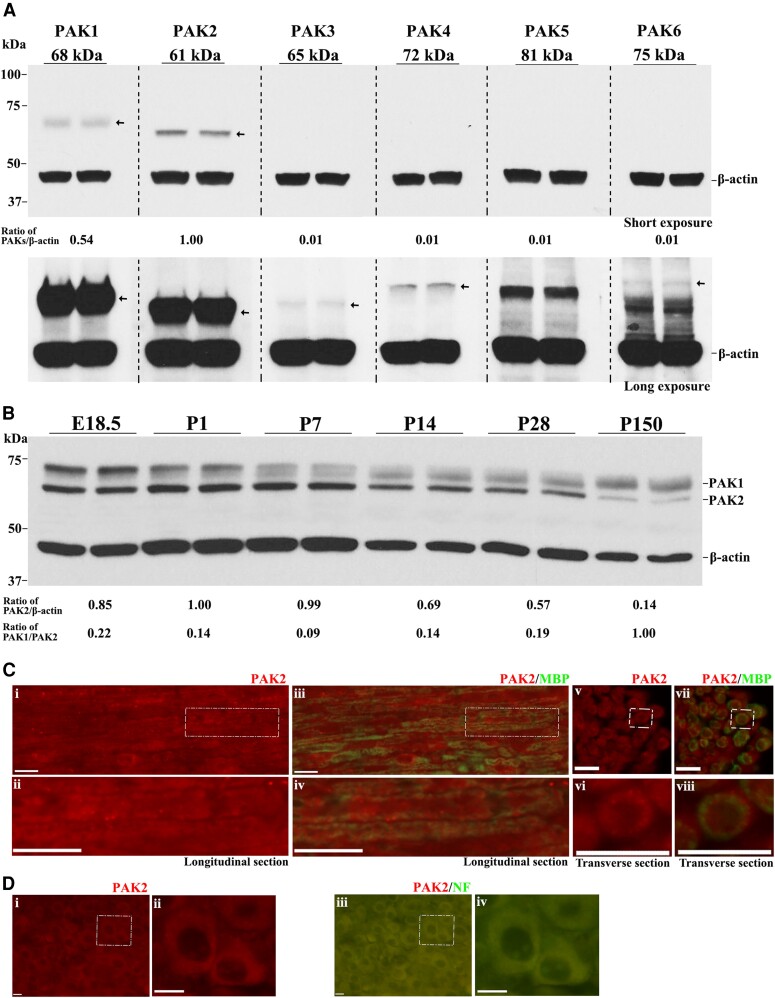
**PAK2 is highly expressed during peripheral nerve development.** (**A**) The expression levels of P21-activated kinases (PAKs) were analysed by western blot in the sciatic nerves of P7 wild-type (WT) mice. Each antibody was tested in duplicates of nerve lysate. PAK1 and PAK2 were detectable in mouse sciatic nerves, while PAK3, PAK4, PAK5 and PAK6 were barely detectable or undetectable. β-Actin served as a loading control. The relative protein level of the PAKs was determined by normalizing to β-actin. The *top* panel was from a short exposure. The *bottom* panel was developed by a long exposure. This experiment was performed three times using different samples. (**B**) Total PAK1 and PAK2 levels were measured by western blot in E18.5, P1, P7, P14, P28 and P150 wild-type mouse sciatic nerves. PAK1 and PAK2 levels were normalized against β-actin levels. PAK2 levels were significantly higher in the developing peripheral nerves (E18.5 to P14) than those in mature ones (P150). PAK1 is highly expressed in peripheral nerves during the embryonic period (E18.5) and gradually decreases during the maturation of the peripheral nerves. For each developmental stage, duplicates of nerve samples were used in each experiment. The experiment was replicated with different samples. (**C**) Longitudinal (**i**–**iv**) and transverse (**v**–**viii**) sections of 3-week-old wild-type sciatic nerves were stained with antibodies against PAK2 (red) and myelin basic protein (MBP) (green, stain myelin). The staining showed PAK2 localization predominantly in myelin. The *top* panels [**C**(**i**, **iii**, **v** and **vii**)] show the original images, while the *bottom* panels [**C**(**ii**, **iv**, **vi** and **viii**)] are the magnified view of the white boxes in the *top* panels. The specificity of the PAK2 antibody was confirmed through knockout mouse tissue (Supplementary Fig. 1). (**D**) A section of the dorsal root ganglia (DRG) from a 3-week-old wild-type mouse was reacted with PAK2 (red) and neurofilament-200 (NF, green) antibodies. PAK2 was localized in the cytoplasm of the DRG neurons. [**D**(**ii** and **iv**)] Magnified view of the white box. Scale bars = 10 μm.

We evaluated PAKs’ expression in the mouse sciatic nerve. PAK1 and PAK2 were detectable by western blot in the wild-type mouse sciatic nerves at postnatal Day (P) 7, while PAK3, 4, 5 and 6 proteins were weakly expressed or not detectable (Fig. 1A). PAK2 had the highest expression among all PAKs. PAK1 expressed higher at embryonic Day (E) 18.5 and P1 but maintained at a moderate level from P7 to adulthood. PAK2 expression was higher from E18.5 to P14, but downregulated in adulthood (Fig. 1B). Immunostaining revealed that PAK2 was present in the myelin of the mouse sciatic nerve and cytoplasm of neurons (Fig. 1C and D). While mice with *Pak1* knockout were asymptomatic with normal morphology in peripheral nerves,^9^ it does raise a question about the *Pak2* function in the peripheral nerve.

### Conditional knockout of *Pak2* in Schwann cells

**Figure 2:**
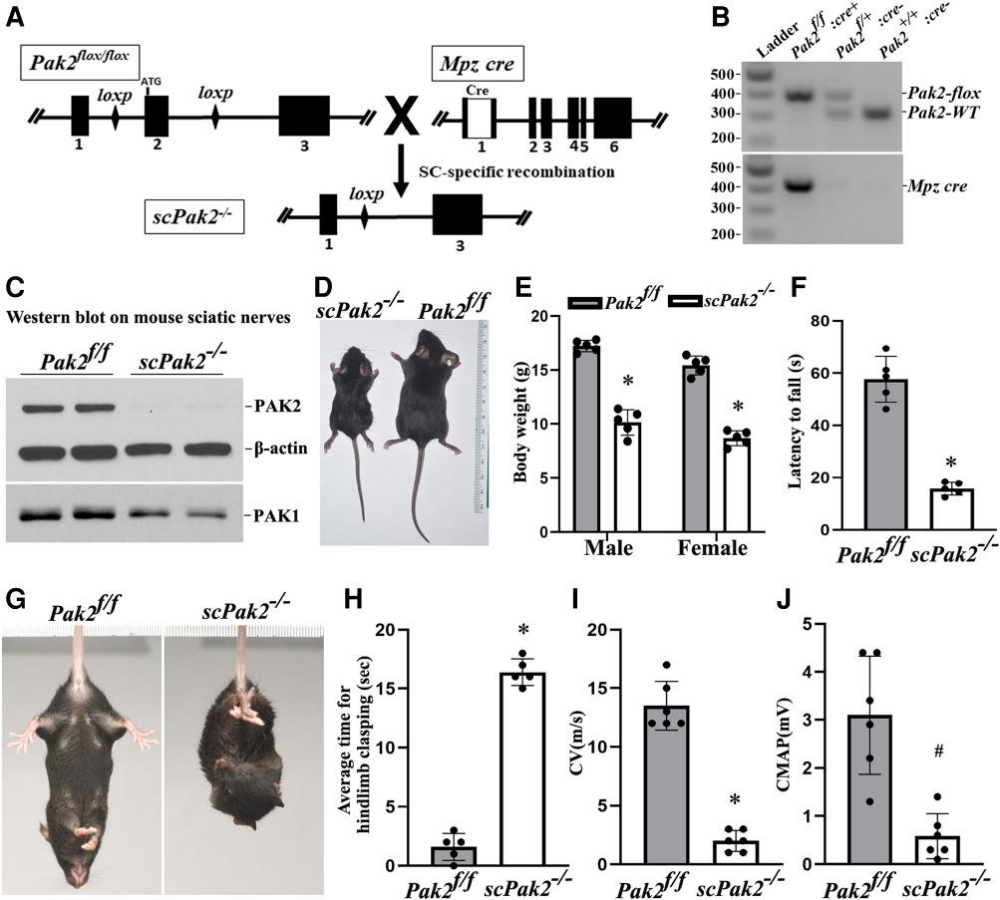
**Schwann cell-specific *Pak2* knockout mouse.** (**A**) Exon 2 in *Pak2* gene was flanked by LoxP. *Mpz* activates Cre recombinase expression in Schwann cells at embryonic Day 14.5. Upon Cre recombination, the exon 2 of *Pak2* between loxP sites was removed to produce Schwann cell-specific knockout mice (*scPak2*^−/−^). (**B**) Mice were genotyped by PCR. The amplified wild-type (WT) *Pak2* allele is 391 bp, and the amplified *Pak2 flox* allele is 306 bp. Lane 1 = DNA ladder (M); Lane 2 = *Pak2^flox/flox^:Mpz-Cre^+^* (*scPak2*^−/−^); Lane 3 = *Pak2^flox/+^:Mpz-Cre^−^* (*Pak2^f/+^*); Lane 4 = *Pak2^wt/wt^:Mpz-Cre^−^* (*Pak2*^+/+^). (**C**) At P21, sciatic nerves with stripped epineurium were analysed by western blot using an antibody against PAK1 and PAK2. In *Pak2^f/f^* nerves, both PAK2 and PAK1 were detectable. However, in *scPak2*^−/−^ nerves, PAK2 expression was hardly detectable, while PAK1 level also exhibited a minor reduction. β-Actin was used as a loading control. The nerves of each genotype were from two different samples. (**D**). One-month-old *Pak2^f/f^* and *scPak2*^−/−^ littermate were placed next to a centimetre measure. (**E**) The body size of the 1-month-old *scPak2*^−/−^ mice was significantly smaller than that of their *Pak2^f/f^* littermates (*P <* 0.001, *n =* 5 in each group). (**F**) Rotarod analysis of motor function at 1 month of age revealed a significant reduction in *scPak2*^−/−^ mice, compared to that in *Pak2^f/f^* mice (*P <* 0.001, *n =* 5 in each group). (**G**) Tail-suspension test showed hind paw clasping in 1-month-old *scPak2*^−/−^ mice, but not in control littermates. (**H**) Quantification of hindlimb clasping test. Data are presented as the average time for hindlimb splaying. One-month-old *scPak2*^−/−^ mice exhibited significantly increased hindlimb clasp compared to that in their *Pak2^f/f^* littermates (*P <* 0.001, *n =* 5 for each genotyping group). (**I** and **J**) Nerve conduction studies showed that conduction velocity (CV) and compound muscle action potential (CMAP) were significantly decreased in 1-month-old *scPak2*^−/−^ mice compared to *Pak2^f/f^* mice (*P <* 0.05, *n =* 6 in each genotype). Mean ± SD. *P < 0.001, ^#^*P <* 0.01.

We crossed *Pak2^f/f^* mice with Schwann cell-specific *Mpz*^cre^ mice (expresses Cre at ∼E14.5) to generate Schwann cell-specific knockout mice. Exon 2 of the *Pak2* alleles was excised during *Mpz-cre* recombination (Fig. 2A). PCR confirmed efficient Cre-mediated recombination (Fig. 2B). Western blot of *scPak2*^−/−^ sciatic nerve confirmed a reduction in PAK2 levels (Fig. 2C), with minimal PAK2 likely from axons and connective tissues. A subtle decrease in PAK1 level was also detected in *scPak2*^−/−^ nerves (Fig. 2C). *ScPak2*^−/−^ pups were born in the expected Mendelian ratio and were similar in size to their wild-type (WT) and heterozygous siblings at birth. However, by P4 weeks, *scPak2*^−/−^ mice had impaired growth (Fig. 2D and E), with death occurring between P6 and 10 weeks.

### *scPak2* ^−/−^ mice manifest behaviour dysfunction

The *scPak2*^−/−^ mice developed tremors by 2 weeks of age and progressive paralysis. At 4 weeks of age, these mice exhibited hindlimb clasping when suspended by the tail (Fig. 2G and H). Rotarod analyses showed significant impairment of motor performance in 4-week-old *scPak2*^−/−^ mice (Fig. 2F). NCS revealed significantly reduced nerve CV (Fig. 2I) and CMAP (Fig. 2J), consistent with abnormal development of myelin.

### Ablation of *Pak2* in Schwann cells arrests myelination

**Figure 3:**
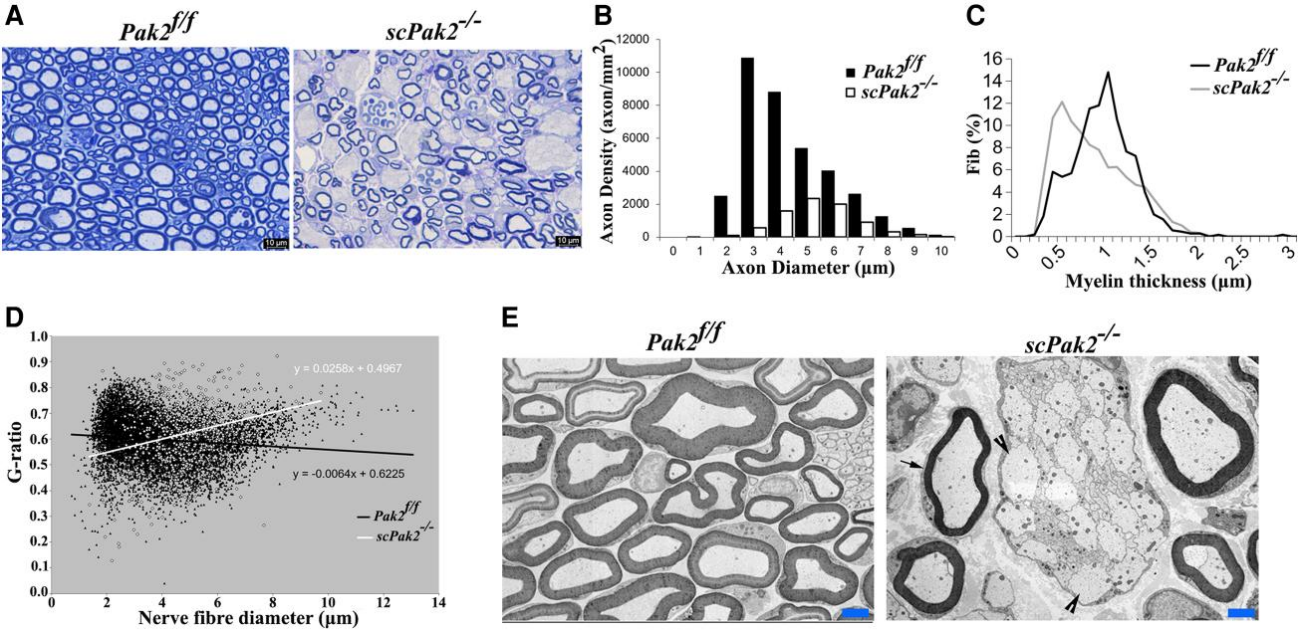
**Morphometric analysis on mouse sciatic nerve.** (**A**) The semithin sections on 1-month-old *scPak2*^−/−^ sciatic nerves showed a reduction in the number of myelinated nerve fibres, which appeared smaller in diameter compared to that in the *Pak2^f/f^* nerves. There were no signs of demyelination, such as myelin debris, broken myelin or excessive macrophages. (**B**) The morphometric analysis confirmed a significant decrease in myelinated nerve fibres of *scPak2*^−/−^ nerves, compared with that in the *Pak2^f/f^* nerves (*P <* 0.001, *n =* 3 in each genotype). (**C**) Myelin sheath thickness was reduced in *scPak2*^−/−^ sciatic nerves. (**D**) A plot of g-ratio against nerve fibre diameter supports the observed reduction of myelin thickness in *scPak2*^−/−^ mice. (**E**) Electronic microscopy images revealed *scPak2*^−/−^ axons either failed to be myelinated (arrowhead) or thinly myelinated (arrow). Scale bars = 2 μm.

We examined nerve morphology on the cross-sections of mouse sciatic nerve under light microscopy and electron microscopy. In *Pak2^f/f^* sciatic nerves, large-diameter axons were well myelinated. However, in *scPak2*^−/−^ mice, we observed axons with thin myelin sheaths and numerous abnormal aggregates of unsorted large-calibre axons that formed axon bundles (Fig. 3A and E, arrowheads). We quantified myelinated nerve fibres using a machine learning-based segmentation.^17^ Our results showed a significant decrease in myelinated nerve fibres and the thickness of the myelin sheath in *scPak2*^−/−^ nerves, compared to those in *Pak2^f/f^* nerves (Fig. 3B and C). Moreover, an increased g-ratio in the *scPak2*^−/−^ nerves confirmed a reduction in myelin thickness (Fig. 3D). These findings demonstrate that the absence of PAK2 in Schwann cells leads to the failure of myelin development.

### Restoration of PAK2 kinase activation, not the protein *per se*, in *scPak2*^−/−^ sciatic nerves rescues dysmyelination

**Figure 4:**
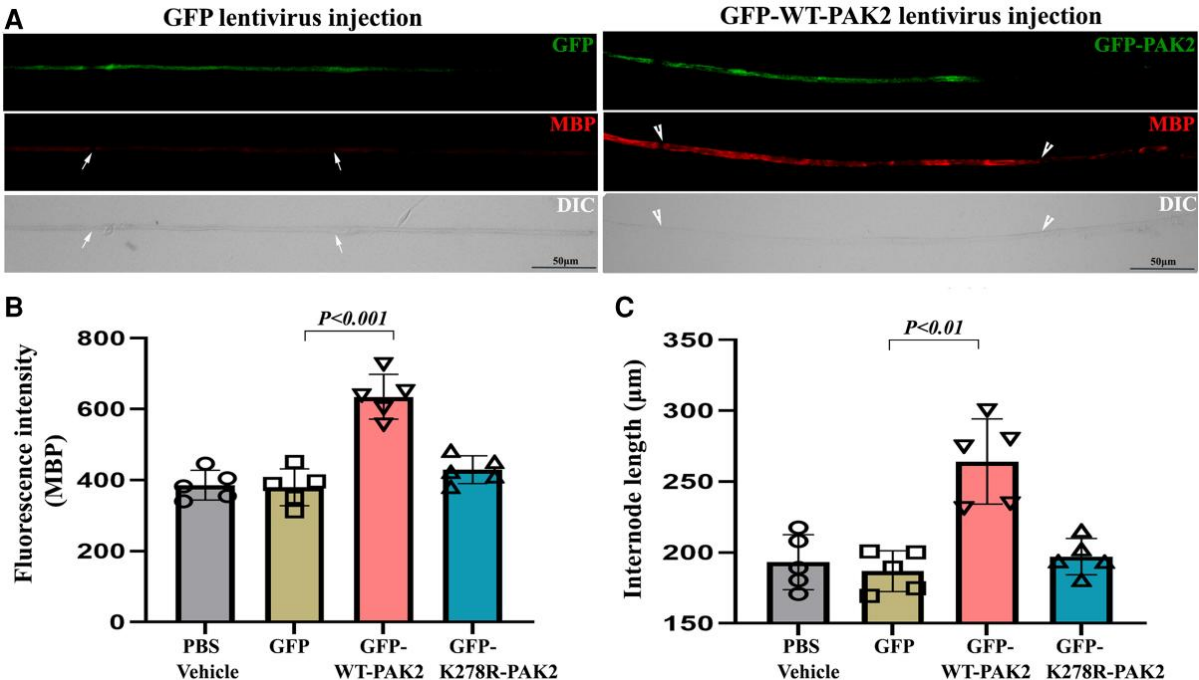
**Intraneural lentivirus delivery restores PAK2 expression in Schwann cells of *scPak2*^−/−^ sciatic nerves *in vivo*.** (**A**) A sciatic nerve from a 3-day-old *scPak2*^−/−^ mouse was surgically exposed and locally injected with 2 μl lentivirus particles at 10^10^ cfu/ml of GFP-only, GFP-WT-PAK2 (wild-type PAK2) or GFP-K278R-PAK2 (kinase-dead PAK2 mutation). The contralateral sciatic nerve was injected with 2 μl PBS as a control. At P30, the sciatic nerves were dissected. After fixation, teased nerve fibres were stained with myelin basic protein (MBP) antibody and imaged. (**B**) The MBP fluorescence intensity of each internode within nerve fibres was quantified and the average intensity for each mouse was calculated. The intensity of MBP was significantly increased in GFP-positive fibres (between arrowheads) from *scPak2*^−/−^ nerves transfected with GFP-*WT-Pak2* lentivirus, but not GFP-positive fibres from *scPak2*^−/−^ nerves transfected with GFP-*K278R-Pak2* lentivirus, compared with fibres (between arrows) in *scPak2*^−/−^ nerve fibres transfected with GFP-only lentivirus (mean ± SD, *P <* 0.001, *n* = 10–20 fibres per mouse, *n* = 5 mice per group). (**C**) Internodal lengths of each nerve fibre were quantified. The average internodal length increased by 40% in *scPak2*^−/−^ nerves treated with GFP-*WT-Pak2* lentivirus (264.2 ± 30 μm for internodes in *scPak2*^−/−^ nerves injected with GFP-*WT-Pak2* lentivirus versus 186.9 ± 14 μm for internodes in *scPak2*^−/−^ nerve fibres injected with GFP-only lentivirus, mean ± SD, *P <* 0.01, 10–20 fibres per mouse, *n* = 5 mice per group). This rescue was not observed in nerve fibres injected with kinase-dead *Pak2* mutation. DIC = differential interference contrast; GFP = green fluorescent protein; SD = standard deviation.

To verify whether the loss of *Pak2* directly leads to dysmyelination, we locally injected 2 μl of 10^10^ cfu/ml lentiviral particles expressing WT-PAK2 or kinase-dead PAK2 into the sciatic nerves of P3 *scPak2*^−/−^ pups. We also injected 2 μl GFP-lentivirus or PBS into the contralateral sciatic nerve as a control. At P30, we teased the sciatic nerves into individual nerve fibres, which were stained with myelin basic protein (MBP) antibodies. We imaged the internodes to confirm the transfection of PAK2 in the lentivirus-injected nerves (Fig. 4A). Quantification of MBP fluorescence intensity (a well-known myelin protein as an indicator of myelination) showed a significant increase of MBP in *scPak2*^−/−^ nerves injected with GFP-*WT-Pak2* lentivirus, compared with the internodes in *scPak2*^−/−^ nerve fibres transfected with GFP-only lentivirus (Fig. 4B). Furthermore, internodal lengths increased by 40% in *scPak2*^−/−^ nerves transfected with GFP-*WT-Pak2* lentivirus (Fig. 4C). In contrast, Schwann cells expressing a kinase-dead mutant of *Pak2* failed to restore MBP expression and internodal lengths in *scPak2*^−/−^ sciatic nerves (Fig. 4B and C). The GFP-only and PBS-injected *scPak2*^−/−^ nerves showed similar pathological changes to those in non-injected *scPak2*^−/−^ nerves. Additionally, PAK2 overexpression did not increase segmental demyelination in wild-type sciatic nerves when compared to nerves with normal PAK2 levels (Supplementary Fig. 2) (0.80 ± 0.2% for nerves with PAK2 overexpression versus 0.73 ± 0.2% for nerves with normal PAK2 levels; *n = 3*, *P >* 0.05). Together, these findings supported the essential role of PAK2 kinase activity in myelination.

### PAK2 is a part of Nrg1- or PrP^C^-driven RAC1/CDC42 signalling in Schwann cells

Nrg1 or PrP^C^ from axons promotes myelination through CDC42, RAC1 and cAMP.^2,3^ Because PAK2 is the effector of the small GTPases RAC1 and CDC42,^21^ we tested whether Nrg1 and PrP^C^ signalling are mediated through PAK2 activity using affinity precipitation and immunoblot assays.

**Figure 5:**
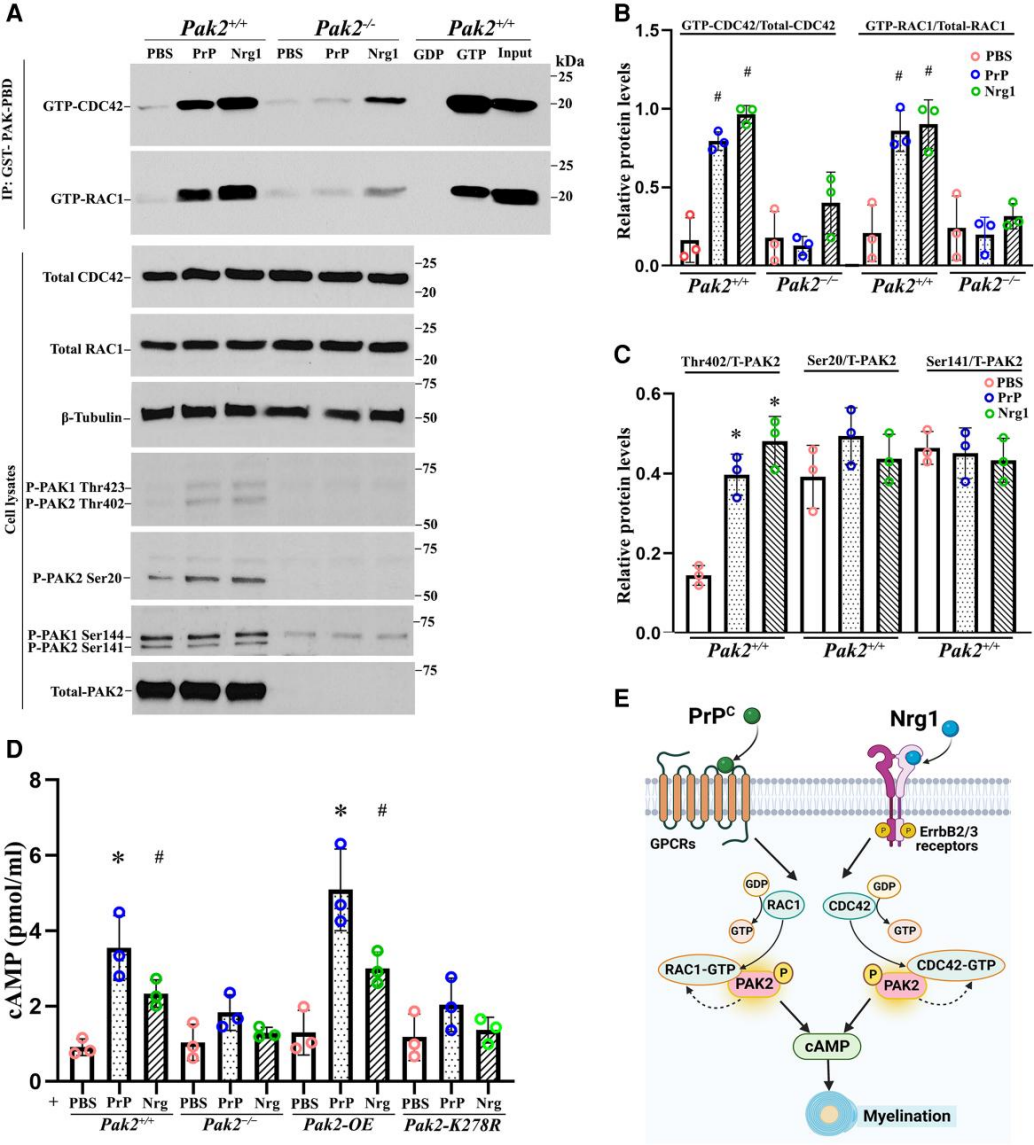
**PAK2 is essential for Nrg1- or PrP^C^-driven CDC42/RAC1 activation and cAMP levels.** (**A**) Endogenous RAC1 and CDC42 activity were measured using an affinity-precipitation assay with recombinant GST-PAK-PBD. Western blots were performed with antibodies for total RAC1, total CDC42, total PAK2 and phosphorylated-PAK2 (Thr402, Ser20 and Ser141) in *Pak2*^+/+^ and *Pak2*^−/−^ Schwann cells stimulated with prion protein (PrP^C^) and Nrg1. (**B**) The levels of GTP-CDC42 and GTP-RAC1 were normalized against total CDC42 and total RAC1, respectively. After 30 min of exposure to PrP^C^ or Nrg1, GTP-CDC42, and GTP-RAC1 levels significantly increased in *Pak2*^+/+^ Schwann cells, compared with PBS-treated Schwann cells (^#^*P <* 0.05). *Pak2*^−/−^ Schwann cells had no or minimal response to the stimulation. (**C**) The level of Thr402, but not Ser20 and Ser 141, significantly increased in PrP^C^ or Nrg1-induced *Pak2*^+/+^ Schwann cells, compared with those in the vehicle (*P < 0.01). Total and phosphorylated-PAK2 proteins were undetectable in *Pak2*^−/−^ Schwann cells. (**D**) Human WT-PAK2 and kinase-dead PAK2-K278R plasmids were transfected into *Pak2*^−/−^ Schwann cells to obtain *Pak2-OE* and *Pak2-K278R* cell clones. *Pak2*^+/+^, *Pak2*^−/−^, *Pak2-OE* and *Pak2-K278R* Schwann cells were treated with PBS, PrP^C^ or Nrg1 for 30 min. The intracellular cyclic adenosine monophosphate (cAMP) concentrations were measured by direct cAMP ELISA assay in cell lysates. PrP^C^ and Nrg1 significantly increased cAMP levels in *Pak2*^+/+^ and *Pak2-OE* Schwann cells, but not *Pak2*^−/−^ and *Pak2-K278R* Schwann cells, compared with that in PBS (mean ± SD, *P < 0.01, ^#^*P <* 0.05). (**E**) A proposed model highlights PAK2 serves as a crucial convergence point for axon-derived Nrg1 and PrP^C^ promyelination pathways, subsequently facilitating the activation of RAC1/CDC42 and cAMP level within Schwann cells. The curved arrows indicate the proposed feedback stimulation from PAK2 to CDC42/RAC1. SD = standard deviation; WT = wild-type.

Schwann cell culture was stimulated by PrP^C^ or Nrg1. The results showed that either PrP^C^ or Nrg1 significantly enhanced the activation of CDC42 and RAC1 in *Pak2*^+/+^, but not in *Pak2*^−/−^ Schwann cells (Fig. 5A and B). This blockage of RAC1 and CDC42 activation by the absence of PAK2 in Schwann cells was not expected and has not been reported. Notice the basal level of RAC1 and CDC42 activation and the total level of RAC1 or CDC42 were not changed in *Pak2^−/−^* Schwann cells (Fig. 5A, PBS lanes). Furthermore, PrP^C^ or Nrg1 significantly increased the phosphorylation of PAK2 at Thr402 (Fig. 5A and C), which is a known indicator of PAK2 activation by RAC1/CDC42.^21^ These findings provide strong evidence that PAK2 activity is required in RAC1/CDC42 signalling activated by promyelinating factors PrP^C^ or Nrg1.

### Nrg1 or PrP^C^ increases cAMP level through PAK2 activity

cAMP is a well-known second messenger to promote myelination in Schwann cells.^5^ Nrg1 or PrP^C^ may elevate cAMP levels in Schwann cells to promote myelination.^4,22^ We performed an ELISA assay to measure cAMP levels. Upon stimulation with Nrg1 or PrP^C^, Schwann cells displayed significantly increased cAMP levels in both wild-type and PAK2-overexpression conditions, but not in *Pak2*^−/−^ Schwann cells (Fig. 5D). Moreover, Nrg1 or PrP^C^ failed to increase cAMP levels in Schwann cells expressing a kinase-dead *Pak2* mutant (Fig. 5D), underscoring an enabling role of PAK2 kinase activity in cAMP signalling. These findings, together with observations in *scPak2*^−/−^ mice above, further support that PAK2 activity is necessary for myelination. PAK2 also appears to be a convergence point for multiple promyelination signalling pathways, as both Nrg1 and PrP^C^ exert their promyelinating effects through PAK2 kinase activity (Fig. 5E).

### Ablation of *Pak2* in neurons does not cause dysmyelination or axon degeneration

**Figure 6:**
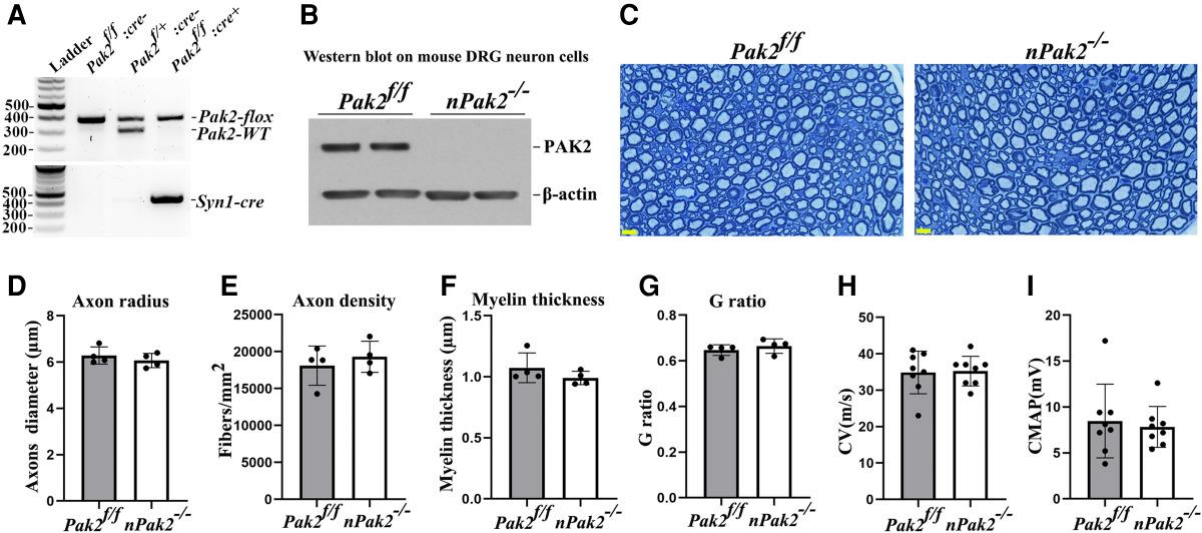
**Neuronal-specific *Pak2* conditional knockout mice.** (**A**) *Pak2^f/f^* mice were crossed with *Syn^cre^* mice to generate a conditional neuronal knockout (*nPak2*^−/−^). The genotype of conditional knockouts was identified by PCR. (**B**) A western blot analysis indicated that PAK2 level was undetectable in neuron cells from *nPak2*^−/−^ dorsal root ganglia (DRGs). The neuron cells of each genotype were derived from two separate samples. (**C**) Semi-thin sections of sciatic nerves showed myelinated nerve fibres were similar between 6-month-old *Pak2^f/f^* and *nPak2*^−/−^ mice. Scale bars = 10 µm. (**D**–**G**) Morphometric evaluations revealed no difference in axon radius, axon density, myelin thickness and the g-ratio between the sciatic nerves of 6-month-old *Pak2^f/f^* and *nPak2*^−/−^ mice (*P >* 0.05; *n* = 4 for both groups). (**H**) Nerve conduction studies showed no significant difference in conduction velocity (CV) between 6-month-old *Pak2^f/f^* and *nPak2*^−/−^ mice (*P >* 0.05, *n* = 8 in each genotype). (**I**) Compound muscle action potential (CMAP) revealed normal values in mutants. Mean ± SD. SD = standard deviation.

PAK2 is expressed in neurons (Fig. 1D). To determine PAK2 function in neurons, we generated a neuron-specific conditional knockout (*nPak2*^−/−^) by crossing *Pak2^f/f^* with *Syn-cre* mice (Fig. 6A and B). Morphometric analysis of the *nPak2*^−/−^ sciatic nerves revealed no significant difference in axon diameter, axon density, myelin thickness and g-ratio when compared to *Pak2^f/f^* nerves (Fig. 6C–G). In line with these findings, both CMAP and CV were not significantly different between *Pak2^f/f^* and *nPak2*^−/−^ mice (Fig. 6H and I). This indicates that promyelination by PAK2 is independent of neurons.

### Promyelination by small GTPases is independent of PAK1

**Figure 7:**
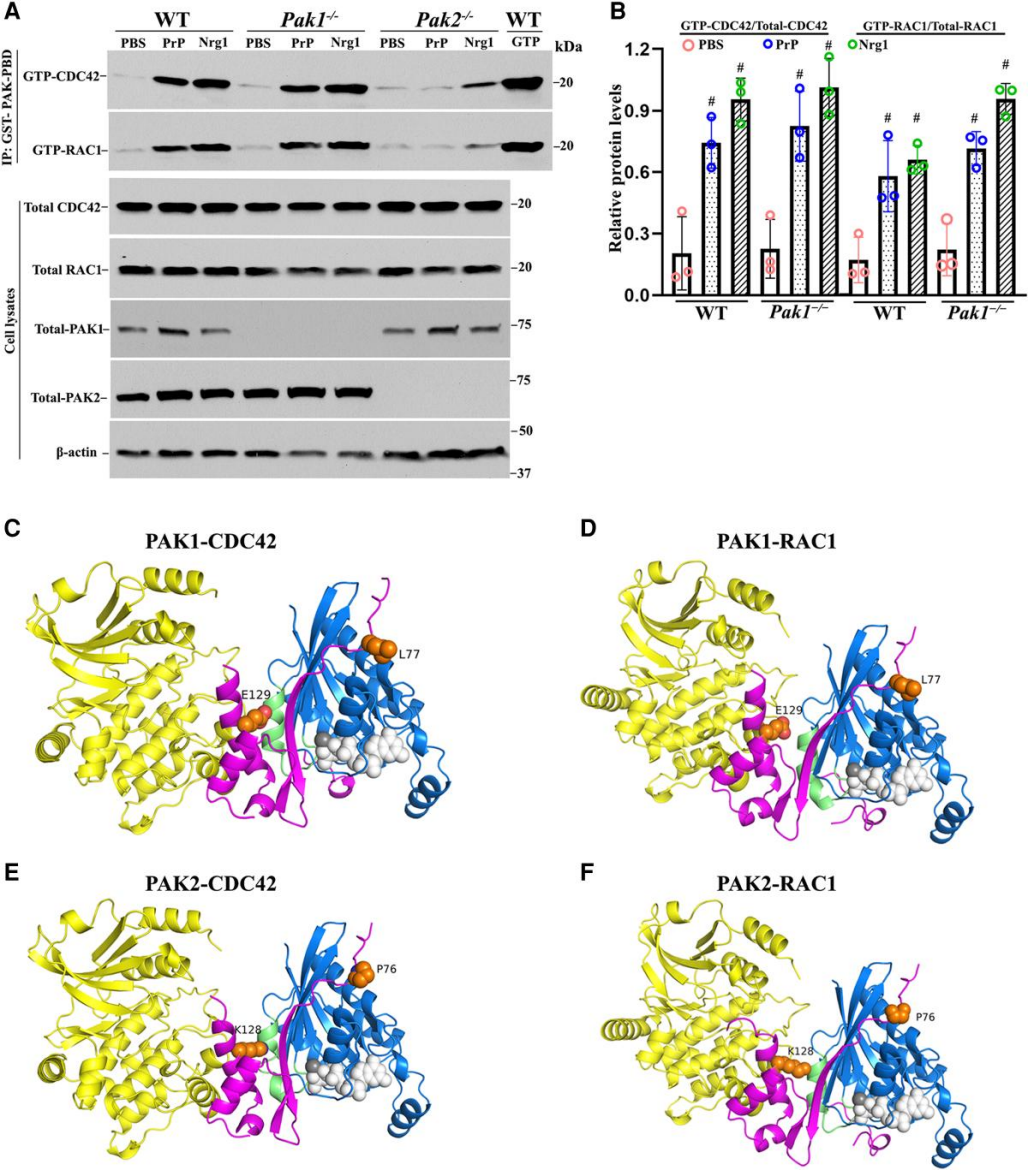
**Comparative analysis of PAK1 and PAK2 in Nrg1- or PrP^C^-driven CDC42/RAC1 activation and protein structure analysis.** (**A** and **B**) The activity of CDC42 and RAC1 were evaluated through an affinity-precipitation assay using recombinant GST-PAK-PBD. Western blot analyses were conducted using antibodies against RAC1, CDC42, PAK2 and PAK1 in Schwann cells of wild-type (WT), *Pak1*^−/−^ and *Pak2*^−/−^, which were stimulated with prion protein (PrP^C^) or Nrg1. Upon stimulation with PrP^C^ or Nrg1, GTP-CDC42 and GTP-RAC1 levels significantly increased in wild-type Schwann cells when compared to the Schwann cells exposed to PBS (^#^*P <* 0.05). On the contrary, *Pak2*^−/−^ Schwann cells showed minimal or no response to the stimulation. (**C**–**F**) AlphaFold-Multimer models of full-length PAK1 and PAK2 with CDC42 and RAC1. (**C**) PAK1/CDC42. (**D**) PAK1/RAC1. (**E**) PAK2/CDC42. (**F**) PAK2/RAC1. Regions with pLDDT (a confidence measure produced by AlphaFold-Multimer per residue) <50 are not shown. These regions are intrinsically disordered regions. The domains are coloured as follows: PAK kinase domain (yellow); PAK PBD regions (magenta); CDC42/RAC1 (blue); CDC42/RAC1 Switch 2 loop/helix (green); GTP from PDB: 5CJP (white spheres); Mg^2+^ ion (grey sphere); residue differences between PAK1 and PAK2 in the PBD region (orange spheres).

RAC1/CDC42 activation depends on PAK2 activity in PrP^C^ or Nrg1 signalling (Fig. 5), which raises a question on PAK1’s roles in the molecular cascade as PAK1 and PAK2 share a high homology in their amino acid sequences. We performed an affinity precipitation and immunoblot assay for PAK1 (Fig. 7) similar to those for PAK2 (Fig. 5). It showed that RAC1/CDC42 activation was not blocked by the ablation of *Pak1* in Schwann cells (Fig. 7A and B).

To clarify the difference between PAK2 and PAK1, we used AlphaFold-Multimer (v3) to model complexes of the full-length proteins (Fig. 7C–F). In each image, the PAK kinase domains are in yellow, the PAK PBD domain (which binds CDC42/RAC1) is in magenta, CDC42/RAC1 is in blue, and the switch two domain of the CDC42/RAC1 is in green. The RAS domain of CDC42/RAC1 is in an active conformation in all four complexes, like that of GTP-bound CDC42 in PDB entry 5CJP (0.3 Å RMSD over backbone atoms). The complex structures are very similar to the crystallographic structures of the PAK1 PBD domain with CDC42 (PDB: 1e0a) and the PAK1 PBD domain with RAC3.

As expected, the structures of the four complexes are very similar. The CDC42 structures are identical in the PAK1 and PAK2 complexes, as are the RAC1 structures with PAK1 and PAK2. Thus, AlphaFold-Multimer does not predict any difference between PAK1 and PAK2 that might affect conformational changes in the switch two, the domain determining the activation of RAS domains.^23^ There are only two residues in the PBD regions that are different between PAK1 and PAK2 and might affect how they interact with CDC42 and RAC1. These are L77 (PAK1) versus P76 (PAK2) and E129 (PAK1) versus K128 (PAK2). Close inspection of the interactions of these residues does not indicate any obvious changes in affinity. E129/K128 does interact with the switch two domains of CDC42 and RAC1, which could be responsible for the differential effects on CDC42 and RAC1 following PAK binding. However, the mechanism for these differences remains to be investigated.

Taken together, these promyelination pathways are dependent on PAK2, but not PAK1, despite high amino acid homology between PAK1 and PAK2.

### Myelin lipids regulate PAK2 activity

**Figure 8:**
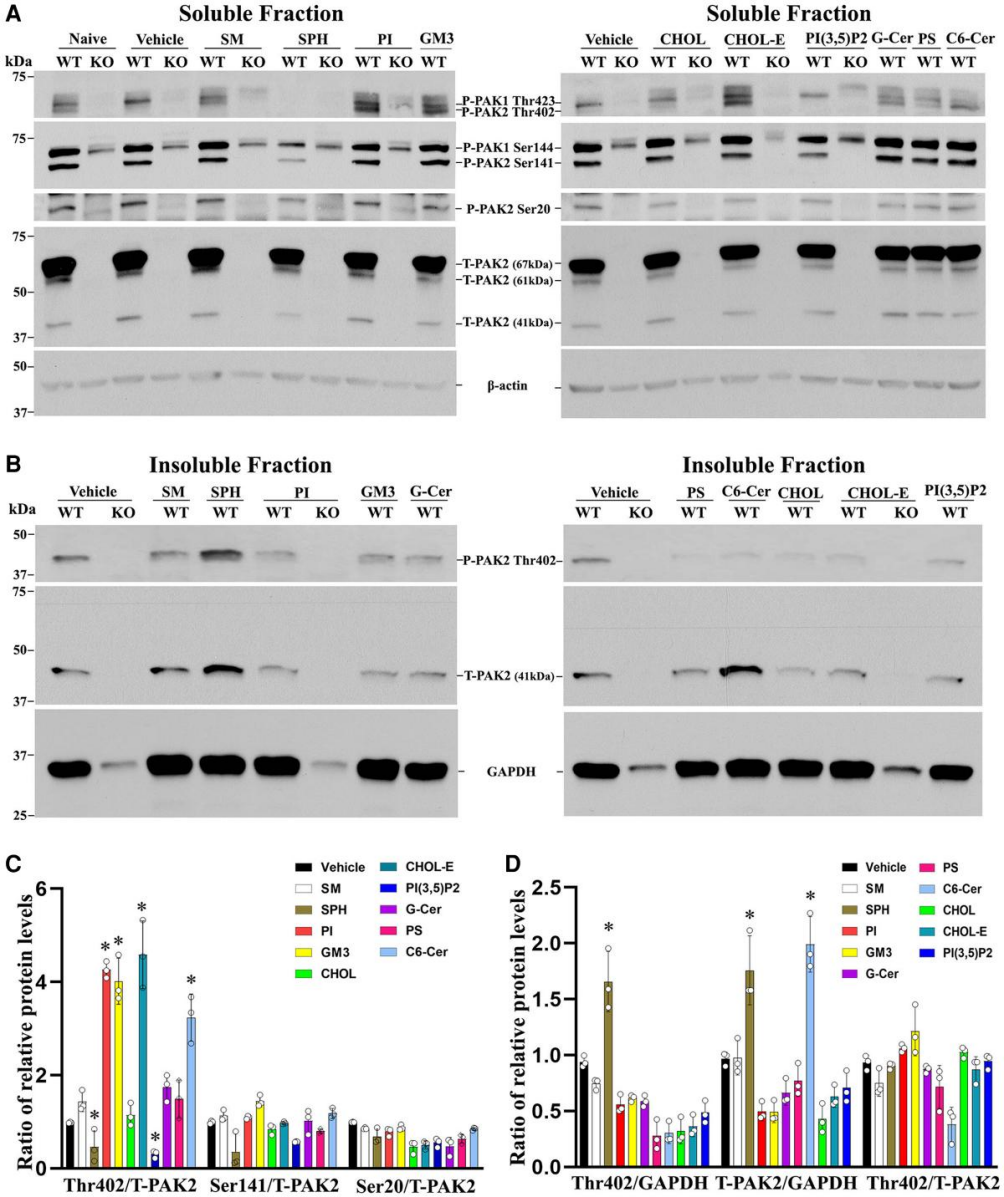
**PAK2 activity is regulated by myelin lipids in Schwann cells.** (**A**) Subconfluent *Pak2*^+/+^ and *Pak2*^−/−^ Schwann cell cultures grown on poly-L-lysine (PLL)-coated wells were starved and stimulated with the indicated lipids (100 μM) or vehicle for 30 min. Cells were lysed in RIPA buffer and the supernatants (soluble fraction) were collected. Total PAK2 and its phosphorylation levels were determined by western blots using the indicated antibodies in *Pak2*^+/+^ Schwann cells, but not in *Pak2*^−/−^ Schwann cells. β-Tubulin was used as loading control. (**B**) The pellets were solubilized by sonication in RIPA lysis buffer containing 1% NP40 and the supernatants (insoluble fraction) were obtained. Western blot of phosphorylated and total PAK2 was performed in the insoluble fraction. The phosphorylated PAK2 proteins (Ser20 and Ser141) were not detectable in the insoluble fraction. GAPDH was used as loading control. (**C**) Densitometric analysis: the Thr402 level, but not Ser20, Ser141 and total PAK2, significantly increased in the soluble fraction of *Pak2*^+/+^ Schwann cells after treatment with cholesteryl oleate, L-α-phosphatidylinositol (PI), C6 ceramide or ganglioside G_M3_, compared to that in vehicles. Conversely, Thr402 level was decreased by sphingosine and PI(3,5)P2. *P < 0.01. (**D**) In the insoluble fraction of *Pak2*^+/+^ Schwann cells, the Thr402 PAK2 level showed a significant increase following sphingosine treatment compared to that by vehicle. Total PAK2 levels were elevated when treated with sphingosine and *N*-Hexanoyl-D-sphingosine. *P < 0.01. C6 Ceramide = *N*-Hexanoyl-D-sphingosine; Chol = cholesterol; CholE = cholesteryl oleate; Gal Cer = Galactosyl(β) Ceramide; GM3 = Ganglioside G_M3_; KO = *Pak2*^−/−^ Schwann cells; PS = L-α-phosphatidylserine; SM = sphingomyelin; SPH = sphingosine; WT = *Pak2*^+/+^ Schwann cells.

Myelin contains a high content of lipids (71%).^24^ Sphingosine has been shown to bind to and activate PAK.^25,26^ We stimulated Schwann cells with 10 different myelin lipids and measured PAK2 activity. Four of the 10 lipids [cholesteryl ester (CHOL-E), phosphatidyl (PI), *N*-hexanoyl-D-sphingosine (C6Cer) and GM3 ganglioside (GM3)] induced phosphorylation at Thr402 of PAK2 in the soluble fraction of Schwann cells (Fig. 8A and C). Interestingly, Sphingosine (SPH) inhibited PAK2 in the soluble fraction but enhanced PAK2 activity in the insoluble fraction (Fig. 8A–D). The two different effects of sphingosine on PAK2 activation have been shown between two different cell fractions from 3T3-L1 mouse pre-adipocytes.^26^

## Discussion

Our study produces several important discoveries that are original. First, it demonstrates PAK2 activity as a novel promyelinating factor. Myelination is essential for the normal functions of the peripheral nerves, including action potential propagation. Disruption of myelination has been found in various peripheral neuropathies, such as those congenital hypomyelination neuropathies, Charcot-Marie-Tooth (CMT) 1A, B, C, D, E, CMT4B, C, D, F and J.^27^ Interestingly, we did observe a decrease of PAK2 activity in a CMT1E (Trembler-J) mouse nerves (Supplementary Fig. 3). Thus, PAK2 could be a potential molecular target to be manipulated in repairing abnormal myelination in these diseases.

Second, the depletion of PAK2 activity blocks signals driven by either Nrg1 or PrP^C^. Thus, PAK2 acts as a converging point indispensable to multiple promyelination pathways. This finding is not only novel but also reveals a broad regulatory effect of PAK2 on myelination.

Abnormal axonal radial sorting in *Pak2*-deficient nerves suggests that the PAK2 effect on myelination takes place at the early stage of myelin development. This is also in line with the highest expression of PAK2 found in developing mouse sciatic nerves. Because deletion of PAK2 blocks RAC1/CDC42 activation (Fig. 5), this finding is also consistent with previous studies that *Cdc42* or *Rac1* ablation in Schwann cells causes severe dysmyelination with abnormal radial sorting.^12,13^ Nrg1 or PrP^C^ functions as key axonal signals that interact with distinct Schwann cell receptors to initiate a cascade of molecular events promoting myelination.^2,4^ Therefore, PAK2 activity is critical for Schwann cell-axon interactions.

While ablation of PAK2 suppressed CDC42 activity, there were still residual activities of CDC42 noticed (Fig. 5, lane 6). How the residual activity occurs is still unclear. However, when Nrg1 binds to the ErbB2/3, it activates not only the CDC42 pathway but also triggers other signallings like focal adhesion kinase (FAK)^2^ that could, in turn, affect CDC42 activity.^28^

The exact mechanisms for PAK2 to drive Schwann cell’s wrapping of axon remain to be determined. Promyelinating factors (Nrg1, PrP^C^) from axons interact with their receptors on the adaxonal membrane (inner mesaxons) of Schwann cells that are anchored on axolemma via various junctions (adherence junctions, tight junctions, gap junctions). These junctions are usually stabilized on the actin network. The actin subject to the regulation of PAK activity,^9^ which could be a mechanism of PAK2 to advance the wrapping of Schwann cell inner membrane. In this context, myelin lipids in the vicinity of PAK2 may directly affect PAK2 activation.

Furthermore, a previous study has shown that CDC42 predominantly affects Schwann cell proliferation while RAC1 regulates Schwann cell process extension.^14^ The latter by RAC1, not CDC42, is mainly driven by the interaction between laminin in the extracellular matrix and β1-integrin on Schwann cells.^29^ PAK2 can also be cleaved by caspase-3 at D212 to release its c-terminal to promote apoptosis via nuclear translocation.^30^ Each of these specific mechanisms could contribute to the phenotypes in *scPak2*^−/−^ mice and remains to be determined. However, this does not alter the conclusion in the current study.

Third, protein-protein interactions are crucial for numerous biological functions, either positively (activation) or negatively (inhibition).^31^ For example, PAK2 activity is known to be stimulated by the binding of small GTPases CDC42 or RAC1. Our study has uncovered a fascinating reciprocal activation between PAK2 and CDC42/RAC1 in Schwann cells, occurring under the drive of axonal Nrg1 or PrP^C^. Small GTPases activate PAK2, but PAK2 activity, in turn, promotes the activation of CDC42 and RAC1 upon the protein complex formation. This reciprocal effect is specific to PAK2, but not PAK1, as ablation of PAK1 made no difference in small GTPase activity (Fig. 7).

This reciprocal effect is also promyelination-specific. The basal activity of CDC42/RAC1 was unchanged in the absence of *Pak2* (Fig. 5), while CDC42/RAC1 activation was blocked in *scPak2*^−/−^ Schwann cells upon the stimulation of Nrg1 or PrP^C^. Our structural analysis did reveal differences of two specific amino acids between PAK2-CDC42/RAC1 and PAK1-CDC42/RAC1 interfaces. It remains to be determined whether the difference is sufficient to explain the reciprocal effect of PAK2-CDC42/RAC1. Nevertheless, these findings do explain the normal peripheral nerve in *Pak1* knockout mice and the severe dysmyelination in *scPak2*^−/−^ mice. Thus, the PAK2-dependence in small GTPase activation underscores the significance of understanding the promyelinating effect conferred by the specificity of the PAK2-GTPase reciprocal effect. This significance is further highlighted by the mutations in the *FRABIN*/*FGD4* gene causing an inherited peripheral neuropathy in humans as FGD4 encodes the small RhoGTPase CDC42-guanine nucleotide exchange factor Frabin and activates CDC42.^32^

Fourth, specific myelin lipid species may enhance or inhibit PAK2 activity. Lipids account for ∼71% of the dry weight of myelin membranes. Myelin has its unique lipid composition with high levels of cholesterol (∼26% by weight) and glycolipids (31%; for instance, cerebroside and sulphatide account for 14%–26% and 2%–7% of myelin lipids, respectively).^33^ During development, most myelin is formed during the first 2 years of postnatal life. In rodents, myelination occurs predominantly during the first month of life.^33^ It has been estimated that the myelin membrane surface area of one glial cell expands ∼6500-fold during its maturation.^34^ The composition of various myelin lipids also dynamically evolves over the course of early development.^35^ How the nervous system senses the myelin maturation to prevent over-myelination is still unclear. Our findings of myelin lipid effect on PAK2 activation provide a potential mechanism allowing myelin lipids to directly regulate PAK2 activity, thereby promoting or curbing myelination.

PAKs have been a ‘hot’ target in therapeutic development through searching small molecule inhibitors of PAKs.^36,37^ Less attention has been given to PAK activators. Our study suggests that PAK2 activity may be manipulated via the delivery of specific myelin lipids or even lipid diet modification.^25,26^

Our finding is likely applicable to the CNS as there is an increase of PAK2 expression in the developing brain.^38^ In fact, *Pak2* haploinsufficiency has been linked to brain development abnormalities and autism aetiology.^38^

Of great interest, mice depleted of PAK1 do not display any evident peripheral nerve phenotype or pathology,^9^ despite a high homology of amino acid sequence between PAK1 and PAK2. Instead, PAK1 hyperactivity has been found to regulate myelin junctions in adult mice with peripheral nerve diseases.^9^ In contrast, the present study demonstrates a prominent role of PAK2 during the early development of the peripheral nerve. We uncovered a novel molecular mechanism differentiating PAK2 from PAK1 through a reciprocal effect between small GTPase and PAK2, not PAK1. Together, each PAK family member exerts distinct biological functions through unique molecular mechanisms.

In summary, this study uncovers a novel promyelinating factor, PAK2. It converges multiple well established promyelinating signalling pathways during glia-axon interactions, such as Nrg1 and PrP^C^. PAK2 exerts its promyelinating effect through small GTPases, which distinct its function from PAK1. Because myelin lipids can enhance or inhibit PAK2 activation, the lipid effect could function as a feedback mechanism to promote or curb myelination, a potential therapeutic approach for future exploration.

## Supplementary Material

awad413_Supplementary_Data

## Data Availability

The authors declare that the data supporting the findings of this study are available within the article and its Supplementary material.
