# Supplementary material for: PAK2 is necessary for myelination in the peripheral nervous system
Source: Brain. 2023 Dec 11;147(5):1809–21. doi: 10.1093/brain/awad413 (PMC11068108; doi:10.1093/brain/awad413)
Supplement: awad413_Supplementary_Data [file awad413_supplementary_data.zip › brain-2023-01379-File010.pdf]

Supplementary Figures and Legends

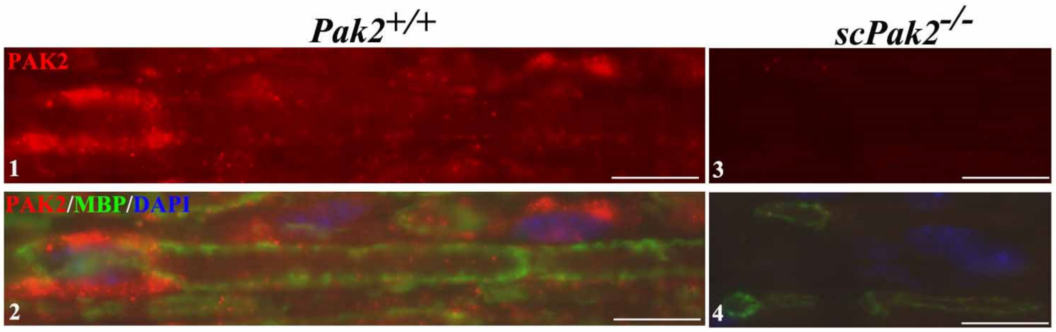

Supplementary Figure 1. Verification of PAK2 antibody specificity. Co-localization of PAK2 and MBP was found in sciatic nerves from wild-type mice. In contrast, PAK2 immunoreactivity was absent in the *Pak2*$^{-/-}$ nerves, supporting the specificity of the PAK2 antibody. Scale bars = 10μm.

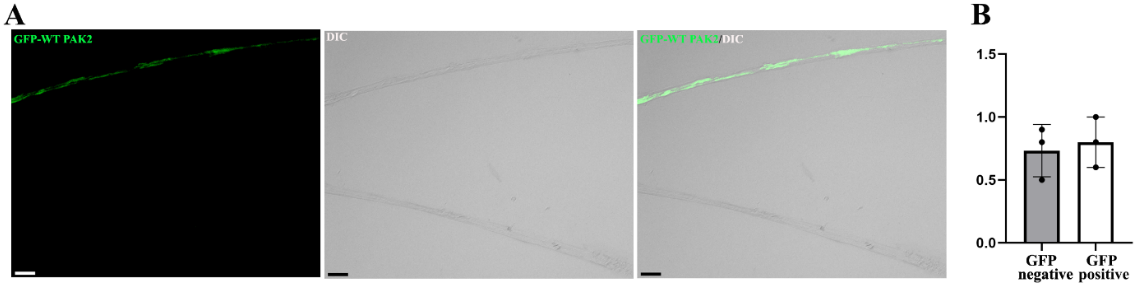

Supplementary Figure 2. PAK2 overexpression does not lead to segmental demyelination in WT sciatic nerves. (A) A sciatic nerve of a 3-day-old wild-type mouse received an injection of 2μl lentivirus particles containing *GFP-WT-PAK2* (equivalent to wild-type PAK2) at a concentration of 10^{10} c.f.u/ml. By P30, the sciatic nerve was dissected, teased into individual fibers, and then imaged. Images with GFP-positive nerves signify the overexpression of exogenous WT-PAK2, whereas GFP-negative nerves suggest the presence of endogenous

PAK2. Scale bars = 20μm. DIC=differential interference contrast. GFP = green fluorescent protein. (B) The percentage of segmental demyelination was quantified. The results showed no significant difference in segmental demyelination between GFP negative and GFP positive nerves (*n=3, P>0.05*).

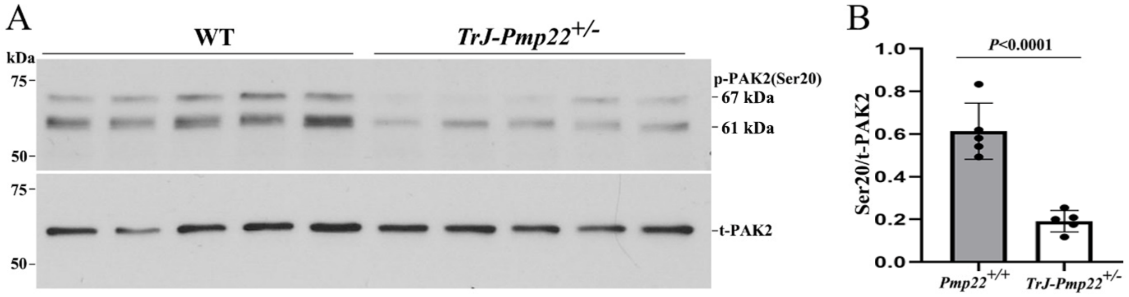

Supplementary Figure 3. PAK2 activity is decreased in *TrJ-Pmp22*$^{+/-}$ nerves. (A) Western blot analysis of Phosphorylated PAK2 (Ser 20) and total PAK2 in sciatic nerves from 3-week-old WT and *TrJ-Pmp22*$^{+/-}$ mice. (B) Ser 20 levels were normalized against total PAK2 levels. The levels of Ser 20 were significantly decreased in *TrJ-Pmp22*$^{+/-}$ nerves, compared with those in WT nerves (Ratio of Ser20/t-PAK2: WT 0.56±0.10 versus *TrJ-Pmp22*$^{+/-}$ 0.20±0.05, $P < 0.0001$, $n = 5$ in each genotype). TrJ =Trembler J.
