# Supplementary material for: PAK2 is necessary for myelination in the peripheral nervous system
Source: Brain. 2023 Dec 11;147(5):1809–21. doi: 10.1093/brain/awad413 (PMC11068108; doi:10.1093/brain/awad413)

Original Blots for Fig 1A.

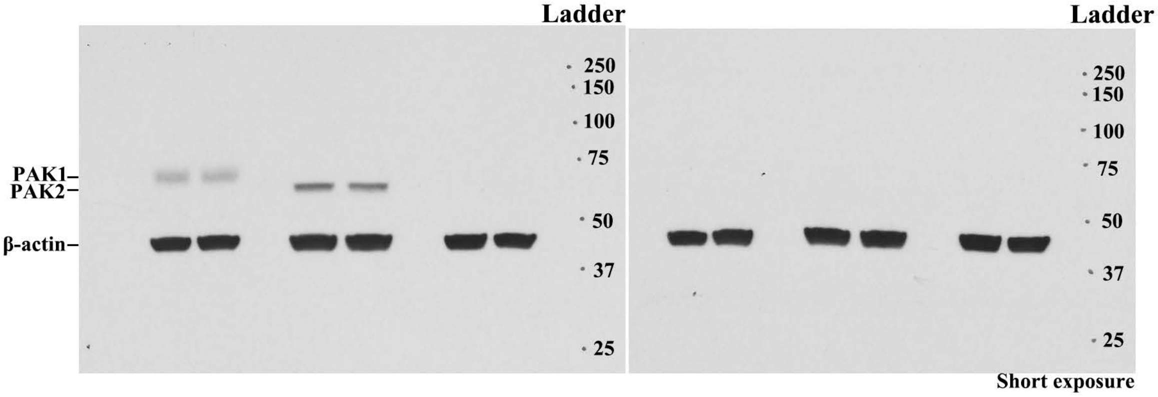

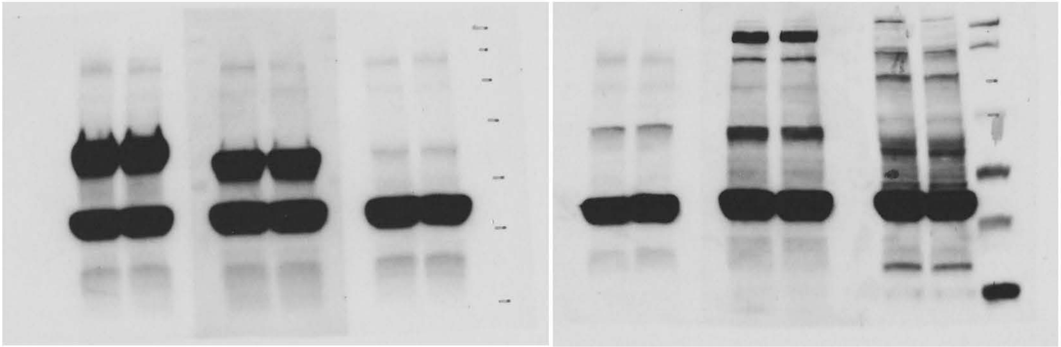

Long exposure

Original Blots for Fig 1B.

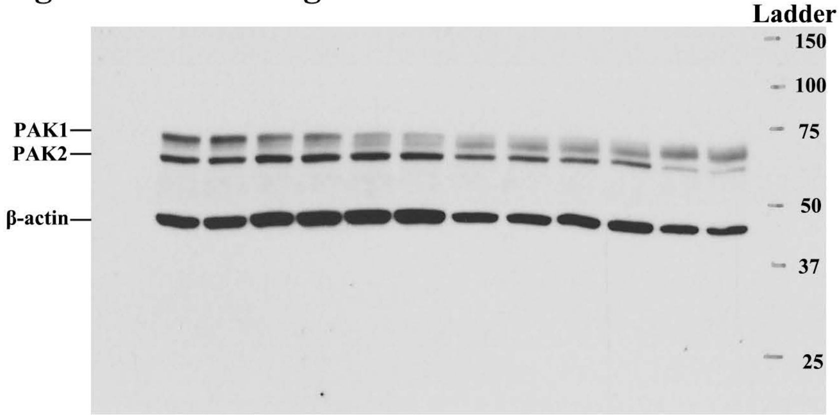

Original gel for Fig 2B.

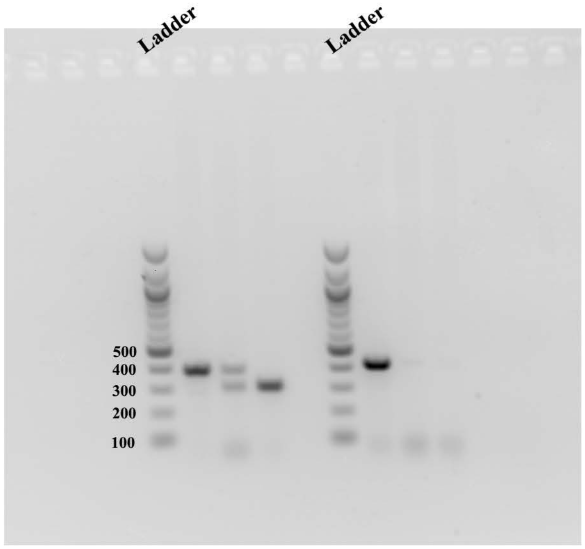

Original Blots for Fig 2C.

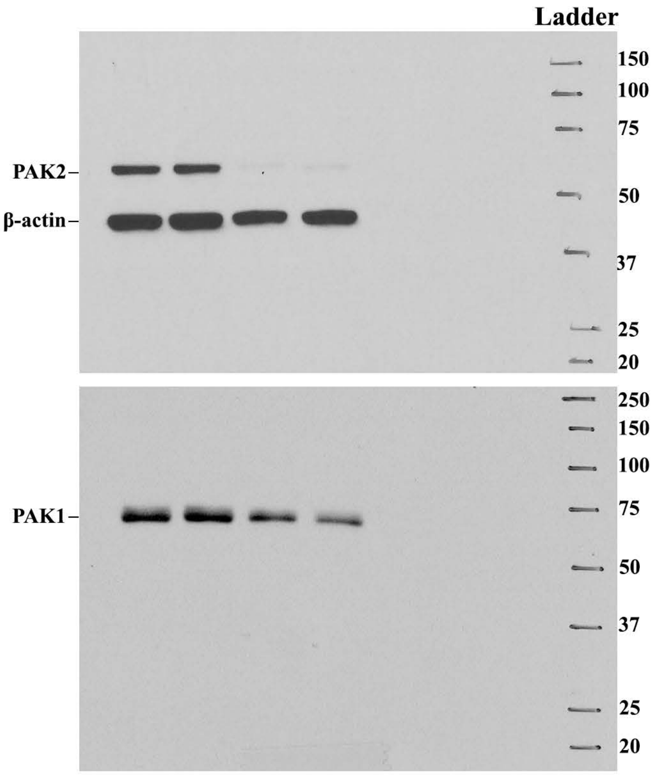

Original Blots for Fig 5A.

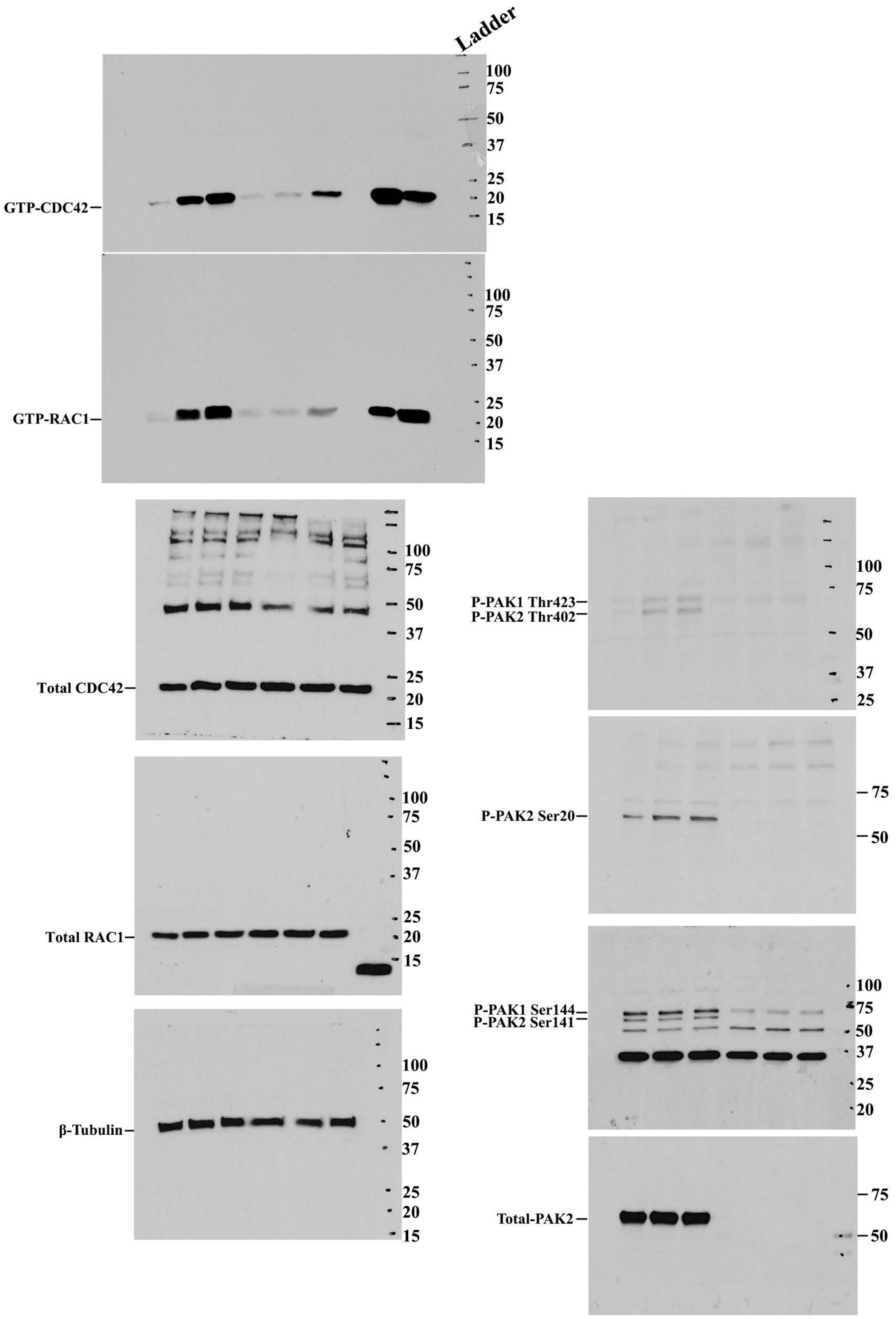

Original gels for Fig 6A.

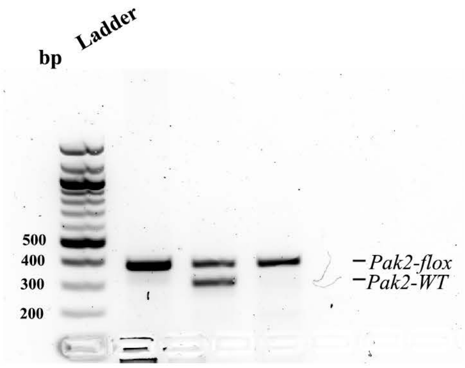

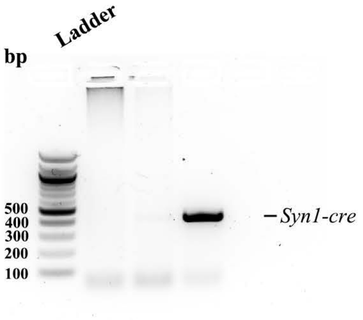

Original Blot for Fig 6C.

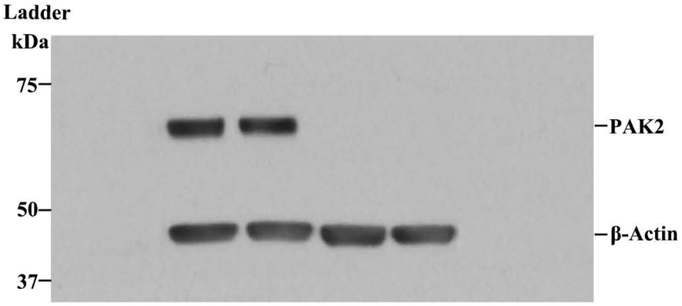

Original Blots for Fig 7A.

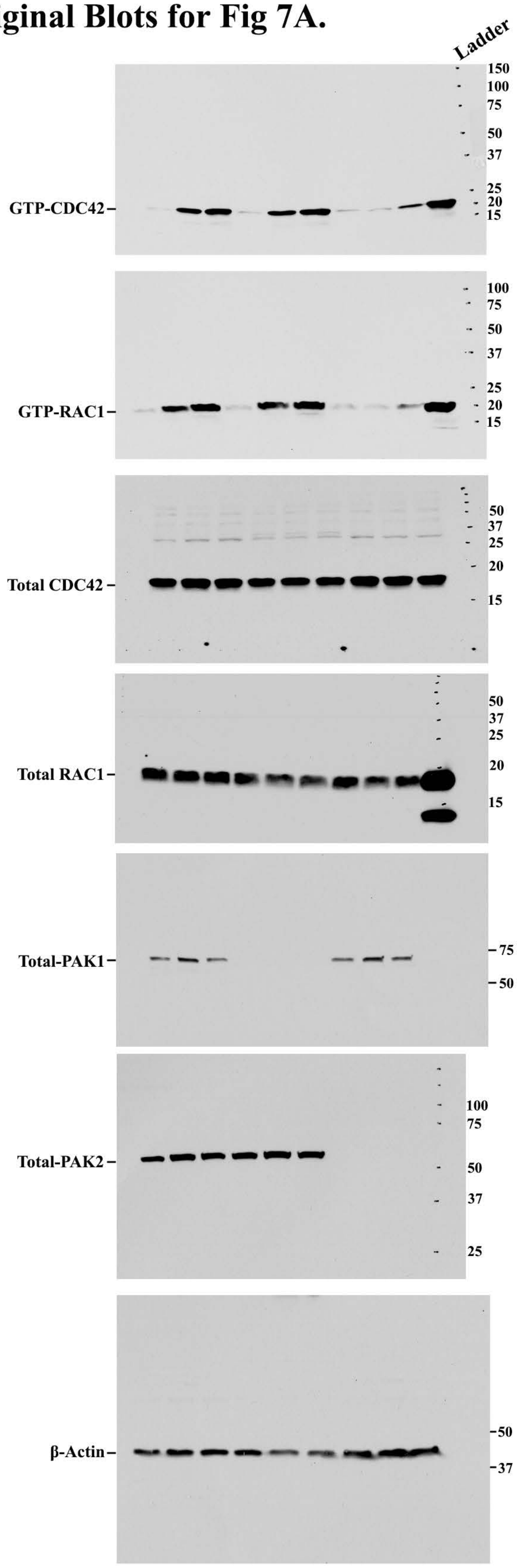

Original Blots for Fig 8A.

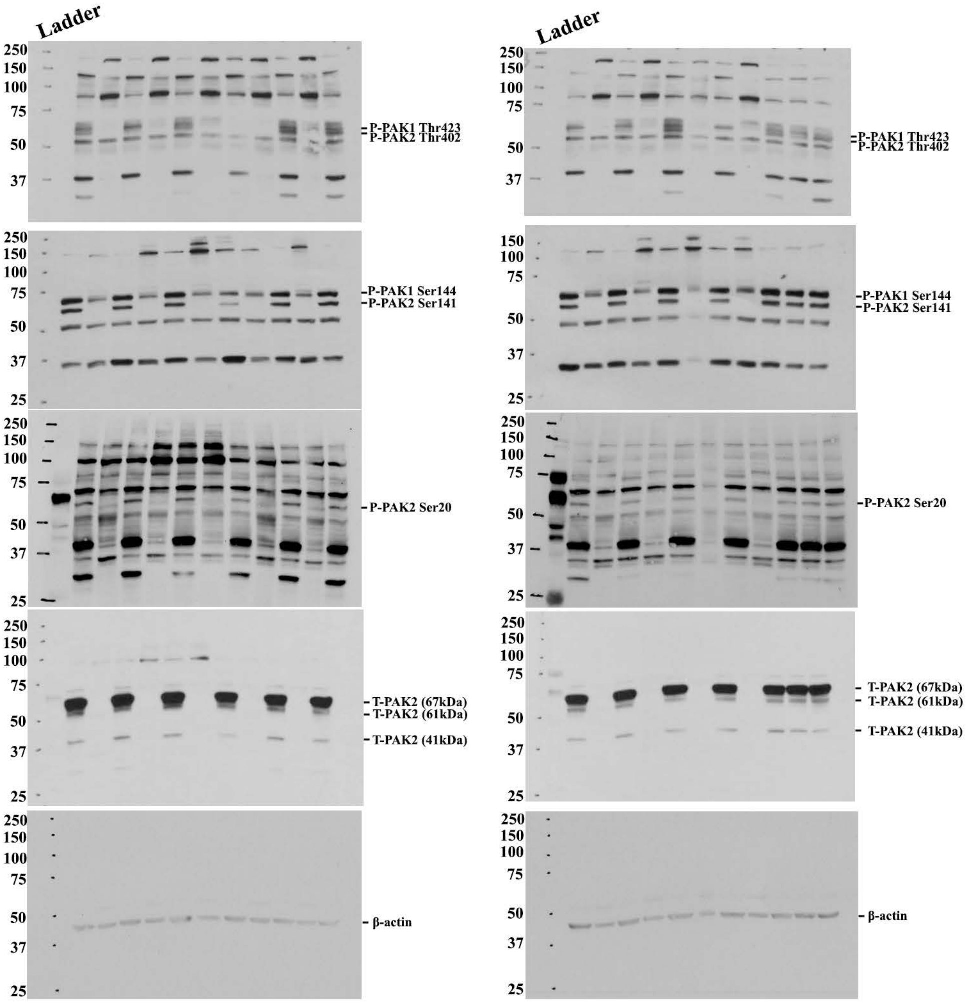

Original Blots for Fig 8B.

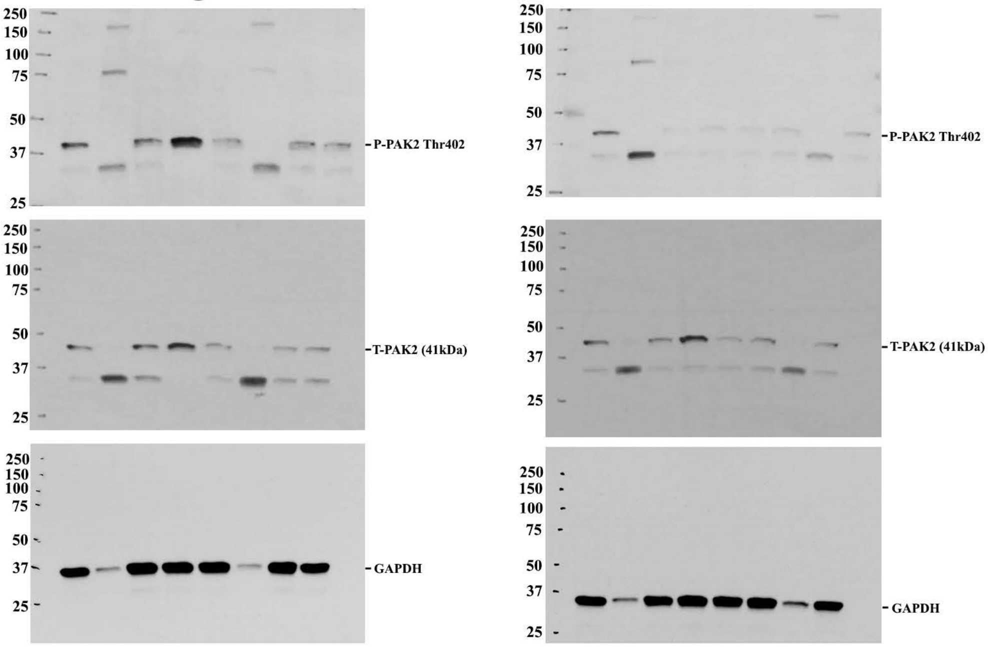

Original Blots for Supplementary Fig 3.

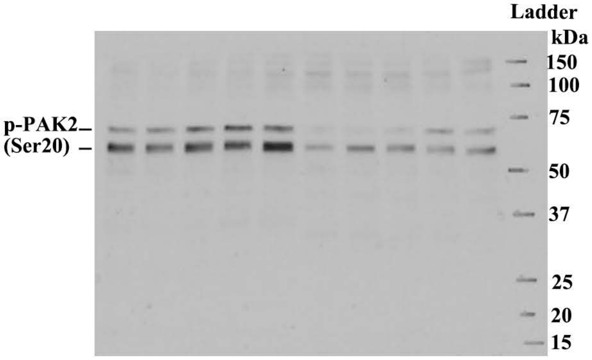

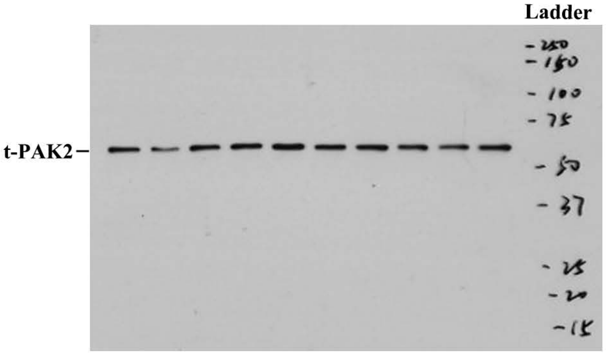

Supplement: awad413_Supplementary_Data [file awad413_supplementary_data.zip › brain-2023-01379-File011.pdf]
